# Case of Enormously Enlarged Stomach

**Published:** 1842-10

**Authors:** John Fondey


*Case of enormously enlarged Stomach.* By John Fondey, M. D.—Professor Horner has handed us the following history of a patient, whose stomach has recently been deposited in the Museum of the University of Pennsylvania. It is of enormous size, probably without a parallel.

Lindsay S. S., aged about twenty-three, was originally from Kentucky (his native place I know not), but for some years past he resided in Tazewell County, Illinois. He was rather a wild youth, and became dissipated in his habits, drinking freely of ardent spirits, yet, although he was in the habit of indulging in very large potations, he was never known to stagger, or indicate in any manner, that he was under the influence of stimulus. In the course of a few years he became the subject of the enormous appetite which constituted so prominent a feature in the history of his disease. Soon after (the exact time I know not), he became insane, yet having lucid intervals, during which he was capable of managing his affairs, and fulfilling his civil and social relations. His insanity was of a harmless character, a prominent feature of which consisted in a propensity to appropriate to himself little articles, no matter how trifling, and he seemed to take as much delight in securing an old nail, could he only do it unobserved, as others would in appropriating to themselves something more valuable, and fitted to excite human cupidity.

The amount of food consumed by him at a meal was very large. He would eat at one meal at least two gallons of mush and milk, and other things in proportion. Sixteen large cups of coffee were about the smallest quantity of this beverage taken by him. Usually this large amount of food was thrown off by his stomach, though sometimes it was retained. He would eat several meals a day, four or five in number, all of them as large as the above in quantity.

By some physicians he was supposed to have a tape worm, by others his disease was attributed to an irritation, or sub-acute inflammation of the mucous membrane, or membranes of the stomach; the correctness of which diagnosis was fully shown on dissection. During his periods of insanity he was strongly inclined to wander, and was placed in the Belleville prison, in consequence of some little theft committed by him in his paroxysm. He was, therefore, kept in some degree of confinement at the house of his brother, from which he managed to escape, and came as far as Canton, near which he received the lynching which resulted in his death.

His disease had by this time produced some effect upon his frame—he was emaciated, though naturally of a good constitution. He travelled in company with an individual of his acquaintance, and when they arrived at Canton he took several keys out of the doors of different shop keepers and put them in his pocket. After they had gone a few miles from Canton, two men overtook them, and charged him with having stolen a trunk from the hotel—he denied it, but confessed that he had taken the keys, and restored them. His companion, however, who was, undoubtedly, the real thief, then affirmed that Scott *had* stolen the trunk, and said there was no use in taking him to Peoria for trial, for they knew him in that place to be a fool, and would only let him go free—that while they had him in their power they had better take the punishment in their own hands. Hereupon they took a teamster's whip, with a long thick silk lash to it, and each in turn whipped him on the back, until the lash was wholly worn out, and then cutting a thick hickory stick, they renewed their blows until they had given him about 250 lashes. He was then released. This was on Monday, December 17th, 1838, and on Sunday evening he arrived at Columbus, Adams County, the place in which I was then located. He had, I understood from some passengers who came in the same stage, eaten three or four enormous meals that day, and seemed rather wild—at times dozing, and complaining much of being half dead with hysterics. His gait was rather unsteady, and he seemed extremely weak. On entering the hotel he requested some water; after it was brought, he laid down, and in ten minutes an individual found him apparently asleep. Some time after, however, on attempting to arouse him, no answer was returned, nor could he be awakened. I was then sent for. When I saw him I found him perfectly destitute of sense or motion. Not a muscle (except those of respiration) moved; his breathing easy, without stertor; pulse variable—now full and hard, then weak, feeble and intermittent; temperature also changeable—at one moment his face flushed, at the next pale—his legs at one moment warm, very soon cold, and then warm again. On flexing the joints, the muscles did not keep the bones in the position thus given them, as in catalepsy. All his symptoms seemed to indicate a prostration of nervous energy, and remedies were prescribed suited to such a condition of the system. He seemed at times dead, but would come to again—this continued on—all remedies were unavailing, and on Monday morning, Dec. 24th, 1838, at 2 o'clock, about eight hours after his arrival at Columbus, he died. After death, however, his eyes remained so clear and prominent, and his skin so elastic and warm, that I doubted whether he was dead, and employed means to bring him to, but on the second day he gave evidence that the principle of life had departed, and he was consigned to the grave.

Four weeks after, his brother arrived, for the purpose of having him examined, preparatory to the trial of those who had beaten him, and I was requested to conduct the dissection. The weather was cold and windy, and we were obliged to examine him in the open air; besides that, we had so little time for examination, that I was not able to make as thorough and satifactory an investigation as I could have wished—in fact I was compelled to leave some organs unexamined, and those which were examined it was found necessary to hurry over, so that if the account of the post mortem appearances is not as full as you would desire, you will, I trust, attribute it to the circumstances in which I was placed.

The skin indicated the reception of a great many lashes, some of them superficial in their effects, others extending down to the bone, as indicated by the immobility of the skin at these points, its hardness, and apparent attachment to the bones. Extensive sugillation was also apparent on the dorsal aspect of the trunk, especially in the lumbar region and the inferior portion of the dorsal. On tearing away the calvarium, the dura mater was found to adhere to it with great tenacity—the parietal foramen rather large, and considerable blood, grumous, yet somewhat fluid, issued from the parietal vein. The vessels of the membranes of the brain, especially those of the pia mater, were rather full of blood—not more congestion, however, I should imagine, than we should look for in one who had labored for some years under insanity. The substance of the brain was very soft, whether from incipient putrefaction, or from the disease, I know not, most probably from the first cause, as no red points could be discovered on making incisions in its substance. Nothing unusual was discovered in the spine, though the examination was here hurried over, from an apprehension that sufficient time would not be left for the investigation of the other organs.

On opening the abdomen, the small intestines were found to be nearly equal in size to the colon. A large portion of them was slit in order to ascertain whether any tape worm was present or not. Nothing of the kind was, however, visible—a little mush seemed to be all that occupied the intestines. No conveniences were at hand to wash the bowel and ascertain the appearance of its internal surface. The spleen and liver were enlarged; want of time prevented an examination of them. There seemed to be a total absence of fat in the parts which were examined. The thoracic cavity was not opened, by reason of the shortness of time.

As regards the appearance of the stomach, you, sir, are better qualified than myself to ascertain the changes of structure it has undergone. I will not, therefore, say any thing on this subject, but merely observe, that it was much more rubescent at the time of the dissection, than when I handed it to you.—*Med. Examiner.*

